# Update on Molecular Diagnosis in Extranodal NK/T-Cell Lymphoma and Its Role in the Era of Personalized Medicine

**DOI:** 10.3390/diagnostics12020409

**Published:** 2022-02-05

**Authors:** Ka-Hei (Murphy) Sun, Yin-Ting (Heylie) Wong, Ka-Man (Carmen) Cheung, Carmen (Michelle) Yuen, Yun-Tat (Ted) Chan, Wing-Yan (Jennifer) Lai, Chun (David) Chao, Wing-Sum (Katie) Fan, Yuen-Kiu (Karen) Chow, Man-Fai Law, Ho-Chi (Tommy) Tam

**Affiliations:** 1 Division of Hematopathology, Department of Anatomical and Cellular Pathology, Prince of Wales Hospital, Hong Kong; nancywilliammurphy@gmail.com (K.-H.S.); cmyuen314@gogglemail.com (C.Y.); 2 Department of Chemical Pathology, Prince of Wales Hospital, Hong Kong; hayliewong1812@gmail.com; 3 Department of Medicine and Therapeutics, Prince of Wales Hospital, Hong Kong; carmen0724@hotmail.com (K.-M.C.); chanyuntat2015@gmail.com (Y.-T.C.); jenniferwy.lai@outlook.com (W.-Y.L.); achaochun3@gmail.com (C.C.); katiewsfan@link.cuhk.edu.hk (W.-S.F.); karenykc@gmail.com (Y.-K.C.); tommybamboo@msn.com (H.-C.T.)

**Keywords:** ENKTCL, EBV, molecular diagnosis, NGS, brentuximab vedotin, immune checkpoint inhibitors

## Abstract

Natural killer (NK)/T-cell lymphoma (NKTCL) is an aggressive malignancy with unique epidemiological, histological, molecular, and clinical characteristics. It occurs in two pathological forms, namely, extranodal NKTCL (ENKTCL) and aggressive NK leukemia, according to the latest World Health Organization (WHO) classification. Epstein–Barr virus (EBV) infection has long been proposed as the major etiology of lymphomagenesis. The adoption of high-throughput sequencing has allowed us to gain more insight into the molecular mechanisms of ENKTCL, which largely involve chromosome deletion and aberrations in Janus kinase (JAK)-signal transducer and activator of transcription (STAT), programmed cell death protein-1 (PD-1)/PD-ligand 1 (PD-L1) pathways, as well as mutations in tumor suppressor genes. The molecular findings could potentially influence the traditional chemoradiotherapy approach, which is known to be associated with significant toxicity. This article will review the latest molecular findings in NKTCL and recent advances in the field of molecular diagnosis in NKTCL. Issues of quality control and technical difficulties will also be discussed, along with future prospects in the molecular diagnosis and treatment of NKTCL.

## 1. Introduction

Extranodal natural killer (NK)/T-cell lymphoma (NKTCL) is a highly aggressive non-Hodgkin lymphoma that is prevalent in Asia and South America and is characterized by a high relapse rate as well as a poor prognosis [1]. ENKTCL is notorious for its resistance to anthracycline-based chemotherapy, including cyclophosphamide, adriamycin, and vincristine. High expression of P-glycoprotein in ENKTCL has been suggested to be the mechanism for its intrinsic drug resistance [2]. Conventional chemotherapy using CHOP (cyclophosphamide, doxorubicin, vincristine, and prednisone) or CHOP-like regimens is associated with an overall response rate (ORR) of less than 60% and a 5-year overall survival (OS) rate of less than 50% [3,4]. Although combination chemotherapy regimens that do not include anthracyclines, such as DeVIC (dexamethasone, etoposide, ifosfamide, and carboplatin), SMILE (dexamethasone, methotrexate, ifosfamide, L-asparaginase, and etoposide), and AspaMetDex (L-asparaginase, methotrexate, and dexamethasone) and radiotherapy have been shown to improve complete remission (CR) rate and OS, the 5-year OS in advanced stage disease remains around 15–25% [5]. Prognostic models have been developed to categorize ENKTCL patients into risk groups to assist with prognosis and patient counselling. One of the commonly used models was the prognostic index for natural killer cell lymphoma (PINK), which consists of the following four independent factors impacting OS: age > 60 years old, stage III/IV disease, distant lymph node involvement, and non-nasal type disease [6]. Subsequently, Epstein–Barr virus (EBV) viral titer was shown to be a biomarker for tumor load and was incorporated into the PINK model, namely, PINK-E [7]. However, these prognostic models do not include tumor biological characteristics nor provide information relevant to targeted treatment decisions.

EBV infection is almost always detected in ENKTCL tumor cells. The detection of EBV-encoded RNA (EBER) aids in the diagnosis of ENKTCL in extranodal sites such as bone marrow and liver, especially when EBV-infected lymphoma cells have a low-grade appearance [8]. However, previous studies on the cytogenetic profile of NK cell lines in non-malignant and malignant NK lymphoproliferative diseases suggested that additional genetic abnormalities may be involved in the multistep transformation of EBV-infected cells into malignant lymphoma [9]. Other studies suggested that NK cells might have acquired CD21 from EBV-infected B cells through “trogocytosis”, an active transfer phenomenon where lymphocytes can exchange their surface molecules [10,11]. Activated EBV-infected T-cells subsequently produce interleukin (IL)-2, IL-9, and IL-10 in an autocrine manner and proliferate [10,11]. The EBV-infected NK cells may first manifest as chronic active EBV infection, but when more genomic alterations such as deletion of tumor suppressor genes and constitutive activation of specific growth or transcription factors occur, the infected NK cells will progress to ENKTCL. The development of molecular techniques, including genomics, transcriptomics, epigenomics, and metabolomics, has shed light on the molecular pathogenesis of ENKTCL, paving the way for the development of new biomarkers and targeted therapy.

## 2. Molecular-Based Research on ENKTCL

**Figure 1:**
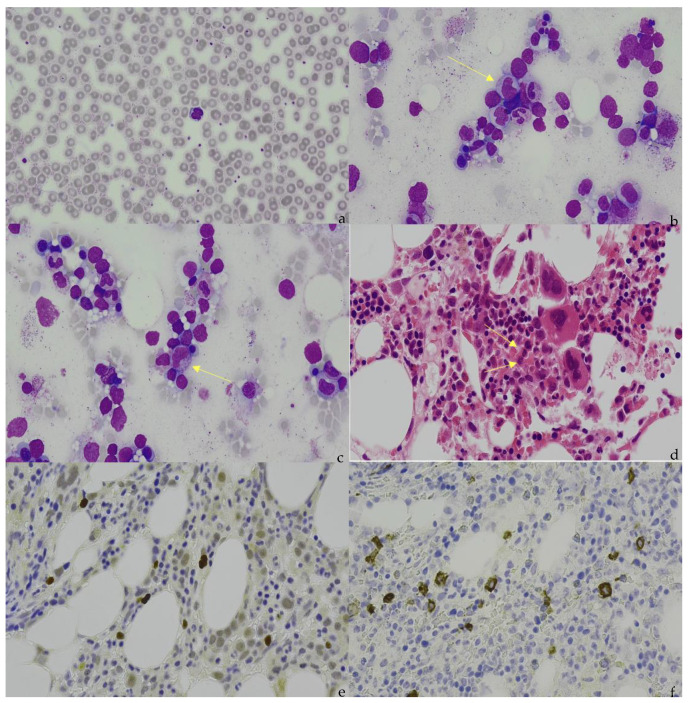
(**a**) Peripheral blood appearance of extranodal NK/T-cell lymphoma (ENKTCL). The ENKTCL cells are of medium size with irregular nuclei, slightly dispersed chromatin, inconspicuous nucleolus, and moderate amounts of granulated cytoplasm. (**b**) (Marrow aspirate), (**c**) (Marrow aspirate), and (**d**) (Trephine). Arrows indicate the infiltration of ENKTCL into the bone marrow. The ENKTCL cells have similar appearance as described in the peripheral blood smear. Trilineage hematopoiesis is represented in this patient. (**e**). Immunohistochemical staining shows that the ENKTCL is positive for CD56. (**f**). In situ hybridization (ISH) staining shows that ENKTCL is positive for EBV-encoded RNAs.

Cutting-edge molecular diagnostic tools have drastically improved our understanding of the molecular pathogenesis of ENKTCL. However, it is clear that these techniques cannot replace the traditional diagnostic tools of light microscopy and immunohistochemistry (IHC) (Figure 1). Nevertheless, the integration of cellular and molecular data is key to developing more comprehensive prognostic models and aid in the development of more personalized treatment approaches with the aim of improving OS and progression-free survival (PFS).

### 2.1. Array-Based Comparative Genomic Hybridization (aCGH)

Microarray is a high-throughput, powerful biotechnology that provides a detailed view of the entire genome, transcriptome, and proteome. The principles of microarray are based on the complementary binding of immobilized oligonucleotides, or antigens, on a chip with fluorescently labelled nucleotides or proteins, respectively, in the samples. A signal is produced post-hybridization and is compared with an unaffected control. This method can produce either quantitative (gene expression) or qualitative (diagnostic) data [12].

Microarrays differ according to the nature of the probe, the solid-surface support used, and the specific method used for probe addressing and/or target detection [12]. Five microarrays have emerged for use in both research and clinical settings, namely, printed microarrays, in vitro-synthesized microarrays, high-density bead microarrays, electronic microarrays, and suspension bead microarrays [12].

The advantage of microarray is that it is easy to use and allows parallel detection of thousands of genes or RNA products in multiple samples. In addition, a wide collection of sub-genome arrays are available commercially, including a collection of promoters, coding regions, transcript 3′ ends, alternative spliced exons, single nucleotide polymorphisms (SNPs), and disease-gene arrays [13]. However, compared with next-generation sequencing (NGS), microarrays are limited by requiring prior knowledge of the genome or genomic features of a particular cancer, and carry a high signal-to-noise ratio due to cross hybridization between similar sequences as well as difficulty in detecting low-frequency mutations [14]. Nevertheless, DNA microarrays have been widely utilized in ENKTCL research and have the potential to be useful in clinical practice. Huang et al. used an in vitro synthesis microarray (Affymetrix GeneChip array) to extensively delineate the molecular pathways and factors involved in ENKTCL and confirmed the role of del6q21 and underexpression of tumor suppressor genes in the 6q21 region, namely, *ATG5* and *AIM1* in these tumors [15]. HACE1, a target of epigenetic inactivation in Wilms’ tumor, was discovered to be markedly reduced. Lastly, expression of the PDGF-α receptor was markedly increased, significantly implicating the associated AKT and JAK-STAT pathways were in the pathogenesis of NKTCL. Interestingly, *TNFAIP3,* encoding an inhibitor of the NF-κB pathway, was underexpressed in the study, highlighting the potential involvement of the NF-κB pathway in the pathogenesis of NKTCL [15].

### 2.2. Next-Generation Sequencing (NGS)

With the ability to sequence multiple fragments in parallel and generate a large amount of data rapidly and inexpensively, NGS offers an unprecedented opportunity to study the human genome, including the whole genome, exome, and transcriptome. NGS enables us to study the molecular pathogenesis of ENKTCL using a multi-omics approach by investigating genomics, transcriptomics, metabolomics, proteomics, and interactomics.

Many NGS platforms currently exist for massive data analysis, including Illumina (Solexa), Roche 454, Ion torrent: Proton/PGM, and SOLiD sequencing. A comprehensive literature search by the authors found that illumine and Ion Torrent panels have been extensively used in the field of NK/T-cell lymphoma.

Illumina acquired the Solexa company, which was the first inventor of a genome analyzer, in 2007. This sequencer uses the principle of sequencing by synthesis. Essentially, the technology involved sequential steps of DNA library construction by fragmentation of template DNA by either sonication, nebulization, or shearing. This is followed by DNA repair, end polishing, and ligation of platform-specific adaptors. Then the DNA library is denatured into single strands and hybridized onto the flow cell, followed by bridge amplification and finally the incorporation of four different nucleotides (ddATP, ddGTP, ddCTP, and ddTTP) onto the complementary strands. With each cycle of incorporation of a nucleotide, a cleavable fluorescent dye attached to the nucleotide is released. The fluorescent signal is subsequently captured by a charge-coupled device. With continual improvements in polymerase, buffer, flow cell, and software, the output drastically increased from a 1 G/run in Solexa GA to 2000 GB/run in NovaSeq 6000 [16]. Moreover, the minimum DNA input amount is as low as 1 ng, and the use of index labels during DNA library construction allows multiple samples to be sequenced at one time. However, the shortcomings of the Illumina sequencer are related to its intrinsic short read length, which poses difficulties in de novo sequencing. In addition, Kircher et al. reported that substitution type miscalls are the dominant source of errors as a result of similar emission spectra of the fluorophores between A and C as well as G and T [17]. Nakamura et al. also identified two major sequence patterns that trigger the sequence specific error (SSE), namely, inverted repeats and GGC sequences. SSE could be a potential cause of false single nucleotide polymorphism (SNP) calls and hinders accurate de novo assembly [18]. Nevertheless, with the features of high throughput and low cost, Illumina sequencers remain the most popular molecular tools for genome analysis in the era of precision medicine.

ENKTCL research using the Solexa/Illumina NGS platforms has produced interesting results. In the field of genomics, Jiang et al. reported that *DDX3X* (RNA helicase) is the most frequently mutated gene, occurring in 20% of study subjects. Moreover, mutations of *DDX3X* seldom overlapped with those of *TP53*, implying that *DDX3X* might be a target of *TP53* or might cooperate with *TP53* as tumor suppressor genes. Importantly, multivariate analysis showed that *DDX3X* mutations and *TP53* mutations, along with International Prognostic Index (IPI) scores, were independent prognostic variables affecting OS and PFS [19]. Dobashi et al. utilized Illumina MiSeq with targeted capture sequencing to study the mutation spectrum in 25 cases of ENKTCL samples in Japan. They found that the most frequently mutated gene was *BCOR*, a chromatin modifying gene, accounting for 32% of the mutations found, followed by *TP53* (16%), *DDX3X* (12%), *FAT4* (8%), and *NRAS* (8%) [20]. Of note, *DDX3X* mutations were less frequent in this study than in the study by Jiang lu et al., which might be due to different ethnic groups and different bioinformatic pipelines. No associations between *BCOR* mutations and OS or PFS were demonstrated [20]. Lee et al. performed whole exome sequencing (WES) and RNA-sequencing on ENKTCL tissues and cell lines using the HiSeq2000 sequencer. Again, genomic analysis revealed enrichment of mutations in the JAK/STAT pathway, tumor suppressor genes, and chromatin-modifying genes. *STAT3* was the most frequently mutated gene (26.5%), followed by *BCOR* (20.6%), *MLL2* (17.6%), and *TP53* (11.8%). Cluster analysis of both cancer tissues and cancer cell lines also highlighted upregulations in genes related to the JAK/STAT signaling pathway, the mechanistic target of rapamycin (mTOR) signaling pathway (downstream of JAK/STAT), and genes related to the PRC2 polycomb complex, which are involved in epigenetic modification (H3K27 trimethylation). Interestingly, genes related to angiogenesis were upregulated only in ENKTCL tissues but not in ENKTCL cell lines. This could possibly be explained by angiodestruction in ENKTCL tissues with compensated angiogenesis, which was not observed in cell lines [21].

In the field of epigenomics, Zhang et al. identified abnormal miRNA expression profiles in ENKTCL cell lines compared with normal NK cells using Solexa high-throughput sequencing. In particular, the level of miRNA-155 expression was abnormally elevated in ENKTCL cell lines and was shown to correlate with the expression of inflammatory mediators such as IL-6, IL-13, and tumor necrosis factor (TNF), suggesting a potential role in promoting the transformation of inflammatory lesions into malignant cells as well as changing the tumor’s microenvironment. Combination treatment with anti-miRNA-155 and doxorubicin increased the sensitivity of ENKTCL cell lines to doxorubicin, which suggests that anti-miRNA-155 may have a role in overcoming drug resistance [22]. Kucuk et al. used the Illumina Genome Analyzer IIx sequencer to study global methylation patterns by methylation-sensitive cut counting (MSCC) [23]. This technique uses the restriction enzyme HpaII, which cleaves CCGG sites only if cytosines are not methylated. Digestion fragments were size-selected and subjected to NGS [24]. The cut sites were then counted and used to infer the number of methylation sites in particular regions of interest. The higher number of cut sites implied fewer methylation sites. Relative to normal NK cells, malignant samples had promoter hypermethylation, gene body hypomethylation, or usually both. In addition, the promoters of tumor suppressor genes (*BIM, DAPK*), negative regulators of the JAK/STAT pathway (*SOCS6*, *ZNFX3*), epigenetics regulator (*TET2*), cytokine receptor (*IL12RB2*), and signaling molecule SHP1 (*PTPN6*) were aberrantly hypermethylated, and mRNA expression of the aforementioned genes was also downregulated as proven by real-time quantitative polymerase chain reaction (qRT-PCR) [23].

Released by Life Technologies at the end of 2010, the Ion PGM and Ion Proton represent groundbreaking molecular tools that utilize semiconductor technology to sequence nucleic acids and are suited for sequencing amplicons or small genomes. To construct a DNA library in Ion PGM and Ion Proton, sample DNA is fragmented and linked to specific adapter sequences and clonally amplified by emulsion PCR on the surface of 3-micron diameter beads (Ionsphere). Enriched particles are then primed for sequencing by annealing a sequencing primer, and subsequently, the Ionsphere with the DNA products is loaded into proton-sensing wells. Sequencing is started by introducing each type of dideoxynucleotide triphosphate (ddNTP) into the microfluidic conduit on the chips sequentially. When bases of that type are incorporated, a hydrogen ion is released, and a hydrogen ion detector translates the released hydrogen ions from each well into a quantitative readout of the nucleotide base [25]. As a result, Ion PGM and Ion Proton are based on “sequencing by synthesis,” but the detection involves counting hydrogen ions that are released during polymerization of DNA and not fluorescence. Because it does not require scanning or measurement of light, an Ion Torrent sequencer such as Ion PGM has a short run time (2 h) and an acceptable read length (~200 bases) [25]. However, compared with the Illumina platform, the Ion Proton platform has a higher rate of misidentifying nucleotide substitutions in the junctional areas of two homopolymers [26]. Possible reasons are related to miscounting of signal intensity when multiple hydrogen ions are released during nucleotide hybridization in homopolymer areas. Choi et al. reported 25 mutations in 21 genes in 5 patients with ENKTL using the Ion Proton platform and identified three recurrent mutations in actionable genes, namely, *KMT2D p.P1194H*, *ARID1A p.P494T*, and *TP53 p.R273P* [27], which were consistent with the results of other studies [19,20,21]. In addition, several other mutations in genes with unknown significance were identified including chromatin modification genes (*SETD2* and *KAT6B*), transcription regulators (*MBD1*, *BCL11B*, *ZNF521*, and *PER1*), genes that regulated cytokines and structural integrity (*CDH11*, *MYH9*, and *SYNE1*), and the DNA damage checkpoint gene (*ATR*) [27]. Montes-Mojarro et al. utilized Ion PGM to investigate the mutational profile of 71 patients with ENKTCL in Latin America and compared the results with other studies. *STAT3* mutation was the most frequent mutation in Latin American cases (23% of cases) [28], compared with *BCOR* mutation in a Japanese study (32% of cases) [20] and *DDX3X* mutation (20% of cases) in a Chinese study [19]. Meanwhile, mutations in tumor suppressor genes (*BCOR*, *DDX3X*, *TP53*) were again confirmed in the Latin American study. In addition, mutations in genes regulating cytoskeleton structure (*MSN*) and transcription (*MGA*) were again identified. Interestingly, mutations in *STAT3*, *BCOR*, and *DDX3X* were mutually exclusive, indicating their role in the pathogenesis of ENKTCL. Lastly, none of the mutations in this study were of prognostic value [28].

### 2.3. Proteomics

Proteomics refers to the analysis of the structure and function of proteins to acquire a global and integrated view of the biology of a cell. Protein expression is regulated at the transcription, translation, and post-translation levels. Compared with the relatively stable state of the genome, proteomics represents a more dynamic state and an ongoing process that underlies the initiation and progression of cancer cells [29]. Apart from traditional methods of studying protein expression, such as flow cytometry or Western blot, a few high-throughput methods are now increasingly used in cancer research, including the search for novel biomarkers, minimal residual disease (MRD) analysis, and deciphering signaling pathways of leukemogenesis; these high-throughput methods include mass spectrometry by electrospray ionization or matrix-assisted laser desorption/ionization (MALDI), protein microarrays, and mass cytometry (CyTOF) [30]. To date, only two groups have utilized proteomics to study molecular aspects of ENKTCL. Xu et al. performed a metabolomic assay on serum samples from 24 ENKTCL patients using liquid chromatography (LC)-mass spectrometry (MS), highlighting an association between aberrant metabolic programming and cancer progression [31]. Interestingly, ENKTCL patients with lower levels of alanine, aspartic acid, glutamate, and succinic acid, which are the amino acids for asparagine synthesis, have a poor response to asparaginase-based therapy and inferior survival. A prognostic score, namely, the ASPM score, was developed based on the above metabolite levels and was shown to independently predict PFS and OS in multivariate analysis [31]. Another group used LC-MS to study proteomic markers of changes in the cerebrospinal fluid (CSF) of ENKTCL patients [32]. After hierarchical cluster analysis by computer software (STRING), the following nine core proteins: HRG, TIMP-1, SERPINA3, FGA, FGG, TF, FGB, APP, and AGT were all higher in ENKTCL patients than in healthy individuals [32]. These proteins have a role in cell adhesion, regulating growth factors and cytokines, tumor invasion, metastasis, and angiogenesis. However, the significance of these proteins in ENKTCL remains to be explored. Nevertheless, proteomic studies offer an additional choice to precisely identify the pathogenesis of ENKTCL.

## 3. Quality Control in NGS

With increasing use of NGS in both clinical and research settings in ENKTCL diagnosis, the first and most important step is to implement a quality control process to ensure data accuracy and precision.

### 3.1. Pre-Analytical Process

The pre-analytical stages of NGS include collection of patients’ clinical details, choosing appropriate sequencing methods and platforms, specimen acquisition, transport, processing, preservation, and storage, as well as NGS library preparation [33]. These steps involve multidisciplinary collaboration between laboratory technicians, surgeons, clinicians, nurses, pathologists, and patients themselves, and play a key role in the success of subsequent steps in NGS analysis. Collection of the patient’s details and communication with patients is also crucial to ensure an appropriate test or sequencing platform is chosen.

Specimen acquisition, transport, processing, and preservation are important steps for reliable and accurate biomarker analysis [33]. Goswami et al. identified five key factors for successful NGS analysis: type of tumor sample, tumor size, tumor location, presence of necrosis, and treatment effect [34]. Specimens collected by resection or excisional biopsy yield the highest NGS success rate. In addition, in order to obtain a minimum of 10 ng recommended DNA input for Ion AmpliSeq Cancer Hotspot Panel, approximately 2000 whole cells are needed and at least 60–100 mm^2^ selected tumor-rich tissue is required [35]. Specimens from tumors in deep-seated organs or lungs, and those from endoscopic biopsies, are generally small, often resulting in tiny, circled tumors (<10 mm^2^) for NGS mutation testing [34]. Of note, head and neck tumors have the highest success rate (100%) whereas bone specimens, which require decalcification with strong acids, have a lower NGS success rate of 62% [34]. Time interval between specimen acquisition and fixation should also be minimized. In general, specimens used for DNA and RNA analysis should be transported to the testing lab within 8 h and 10 min, respectively [36]. Tissue processing and preservation are critical steps as well to ensure sufficient quantity and quality of specimens for optimal morphological diagnosis and NGS analysis. Formalin is a commonly used tissue fixative in most anatomical pathology laboratories due to its superior ability to preserve morphological details by fixing both cytoskeletal and soluble proteins [37]. However, formalin fixation can potentially lead to cross-linking between nucleic acids, DNA denaturation, DNA fragmentation, and creating artificial mutation [38]. Methanol-based fixation is associated with higher yield of DNA fragments and shows better correlation than formalin-based fixation in calling copy number variations in snap-frozen biopsies. Thus, methanol is suggested as a better preservation method for subsequent NGS analysis than formalin-based fixation. However, more studies are required to evaluate the effect of long-term methanol fixation on DNA quality and quantity as the only available study utilized samples collected no more than 6 months previously [39]. Extracted DNA should be stored at −20 °C and RNA at −80 °C to maintain sample integrity. Sequencing libraries and PCR products may be stored at −20 °C [40].

### 3.2. Analytical Process

Whilst NGS is a promising molecular technique in the diagnosis, prognostication, and treatment of ENKTCL, it generates a massive amount of bioinformatic data compared with conventional Sanger sequencing. Because of the huge amount of data, a tightly regulated analytical sequence and process have to be implemented and maintained to ensure the data and results obtained are accurate and precise. The data analysis pipeline (also called bioinformatics pipeline) typically involves the following four major steps: base calling, read alignment, variant identification, and variant annotation. The complex analytical process requires different bioinformatics software and tools that are available commercially as well as on the internet. Essentially, the very first step of NGS involves translating the fluorescence generated during nucleotide incorporation into different base pairs, i.e., base calling. After filtering out bad reads, eliminating the sequencer’s bubble effects, and trimming reads at front and tail by bioinformatics software such as AfterQC, various parameters can be determined in order to conduct the subsequent analysis. “Per base sequence quality score” looks at the probability that a base has been identified incorrectly. The Illumina platform introduced the Q score (Q = −log10(e)) where “e” is the estimated probability that a base is called incorrectly. A good quality read, especially for mutation detection, should ideally have a score of Q30 or above. Of note, sequences at the start and end of each read may have a lower Q score due to ineffective synthesis by DNA polymerase at the beginning and end of the reactions. Santani et al. recommended >80% of bases should be Q ≥ 30 [41]. A sufficient quantity of reads and depth of coverage are also important. Coverage refers to the number of times each nucleotide is sequenced [42]. Regions with low depth of coverage have a higher false negativity rate and should be eliminated from analysis. In general, whole-genome sequencing (WGS) and targeted gene sequencing should at least achieve 30x and 500x depth of coverage to ensure read quality. However, the total number of required reads and depth of coverage vary between laboratories as different laboratories have different analytical sensitivity and different pre-analytical requirements for minimum tumor content. The appropriate number of reads and depth of coverage should be determined in the NGS validation step, which is outside the scope of the current review. “Per sequence GC content” distribution curve, which plots the percentage of GC content vs. number of reads, is also an essential parameter. Theoretically, in a random DNA library, the distribution of GC content should be close to normal distribution. Abnormal peaks or a shift of the actual curve relative to the theoretical curve may indicate another source of contamination such as DNA from bacteria and rRNA contamination. These sources of contamination must be eliminated before further downstream analysis. “Uniformity of coverage” which measures variability in sequence coverage across target regions is an important parameter in NGS. Certain regions in the gene sequence such as at the edges of targeted regions, regions with high GC content as well as regions with homopolymers, may have lower coverage depth which affects the quality of the data. Strategies have been devised to tackle this issue in hybridization-based enrichment techniques, such as a repetitive bait design process, in which a less-dense bait tiling is tested first and underperforming regions are then optimized [41]. Moreover, one can consider extending the baited region beyond the actual region of interest to increase the coverage at the edges of targeted regions [41]. One should also be aware of the “mapping quality score” which is basically the probability that a read is aligned with the wrong position in the human genome. The probability is calculated as P = 10^(−q/10)^ where q is the mapping quality. Multiple mappings are typically seen in repetitive regions especially if using sequencers that produce short reads such as HiSeq2000 (2 × 100 base pairs in length). PCR duplicates should be removed prior to analysis to ensure that duplicated sequences, which may sometimes contain artificial mutations, will not be overrepresented [42]. During library preparation for NGS, we first fragment the DNA, e.g., by sonicator or enzymes, and then ligate the adapters to both ends of the fragments and amplify the fragments with the adapters. During PCR amplification, multiple copies of the same DNA fragments will be generated and thus a certain number of PCR duplicates are unavoidable. However, abnormally high proportions of PCR duplicates may suggest possible bias generated during DNA library preparation especially in sequences with low GC content and short read lengths. Several bioinformatics tools are now available to remove DNA duplicates, including Ion AmpliSeq Cancer Hotspot. To increase the confidence in calling variants, confirmatory tests by Sanger sequencing or a combination of two sequence platforms such as WES and WGS should be performed [43].

### 3.3. Post-Analytical Process

**Figure 2:**
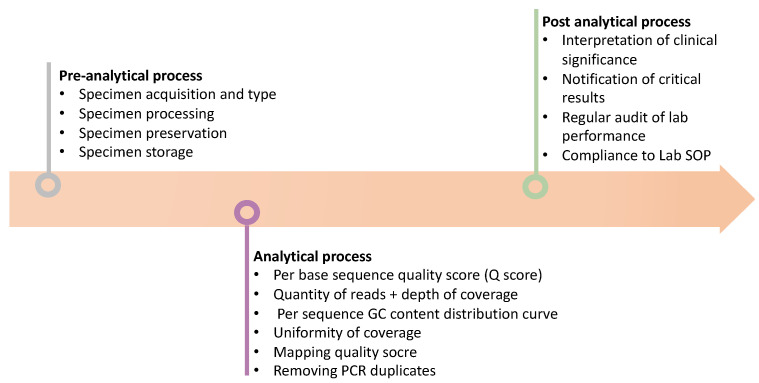
Schematic diagram briefly illustrates the procedure to qualify the NGS results. Firstly, perform the pre-analytical analysis before processing NGS, such as observing the specimen type and preservation etc. Secondly, observe the quality of the NGS results, such as Q score, read depth, and coverage rate etc. Finally, interpretate the NGS results based on the guideline and the clinical indication.

Post-analytical processing involves converting the results into a written report, immediate notification of critical results to the responsible clinician, assessment of the significance of the results based on established reference values, transmission of reports without delay to the requested location, and finally a regular audit of laboratory performance [44]. A molecular laboratory should have a standard operating procedure (SOP) document to tightly regulate the aforementioned processes and ensure traceability of records. In general, the turnaround time from sample collection to result reporting should be around 1–2 months and an annual review as well as update of the previous report based on the most recent molecular findings are needed [41]. A summary of these processes is shown in Figure 2.

## 4. Potential Sources of Error of Using NGS in ENKTCL Diagnosis

ENKTCL is most commonly present in the upper aerodigestive tract, particularly the nasal cavity. One potential source of error in NGS testing lies in the sample preparation steps in formalin-fixed and paraffin-embedded (FFPE) samples from the nasal cavity. Formalin, a liquid containing 37–40% formaldehyde and 10% methanol, can cause artificial DNA cross-linking, DNA fragmentation and degradation, deamination of cytosine (resulting in either a C→T or a G→A on the sense strand), and hydrolysis of N-glycosyl bonds [45,46,47,48]. These artificial sequence artifacts could interfere with the detection of low-frequency mutations. Lin et al. reported the highest level of baseline noise level could be up to 1.3% in the EGFR gene in cancer-free FFPE samples, attributable to artificial cytosine deamination [49]. Williams et al. demonstrated that 97% of artificial mutations in FFPE samples occurred at guanosine or cytosine positions and resulted in C→T or G→A transitions [45]. Several methods have been devised to tackle this artifact, including duplicate experiments, pre-treatment of FFPE samples with uracil-DNA-glycosylase, and heating the FFPE samples at a high temperature and alkaline pH to increase the yield of DNA from FFPE samples [50].

DNA fragmentation is another potential source of errors during the NGS study for ENKTCL. During library preparation, ultra-sonication as a non-biased manner has been widely used to prepare DNA fragments of an appropriate size. However, during ultra-sonication, the physical scattering of DNA solution leads to a loss of DNA sample. In view of this, enzymatic fragmentation with DNA endonucleases has been developed to minimize the loss of DNA, especially for nano-quantity samples. The downside of enzymatic fragmentation occurs when the DNA fragments produced by this method are too long to be sequenced by NGS.

Barcode contamination is another issue to be paid attention to. During NGS data analysis, some non-target fragments that came from the last round of NGS samples but with the same barcode utilized are detected. These non-target fragments will be misread and contaminate the target data. Thus, different barcodes should be used after each round of sequencing.

## 5. Designing NGS Panel Content

NGS yields a tremendous amount of genetic information. However, the complexity of genetic information can sometimes hamper the diagnostic process, especially when some gene mutations could just represent a constitutional change. Thus, designing the NGS panel must take several factors into consideration. In a research setting, NGS could be useful in discovering novel mutations. A more comprehensive panel that includes coding regions of several hundred genes or a pan-cancer gene panel will be more useful. However, in clinical settings, genes with known mutation hotspots and those that carry diagnostic, therapeutic, and prognostic information will be more desirable. Meanwhile, we have to consider the quality of the sequencing data as well as the financial cost [51]. As more genes are sequenced, the quality of sequencing will be compromised in terms of read depth and depth of coverage. In addition, it will involve more complex analytical issues and a longer turnaround time. Moreover, the technical limitations of NGS have to be considered when designing a gene panel. Due to the short read lengths of NGS technology and the complexity of the genome, challenges arise from the detection of copy number variations (CNV) of the disease-related genes. Different approaches have been recently devised to detect CNVs, namely, paired-end mapping, the split read-based method, the read depth-based method, and the assembly-based method [52]. However, a validated approach/guideline to detect CNVs is still lacking. Thus, one has to take into consideration whether your laboratory has sufficient bioinformatic support when designing a gene panel involving CNVs.

An exhaustive literature review by the authors identified the five most recently published large-scale NGS studies on ENKTCL as shown in Table 1 below. Mutated genes that were identified in ≥2 patient samples/cell lines are listed in the table. This table of genes is not validated in the author’s laboratories, but each genetic mutation could possibly be a future topic in later research. At the time of writing this review, only *DDX3X* and *TP53* mutations have been shown to have a prognostic impact [19].

## 6. Future Directions in the Molecular Diagnosis of ENKTCL

The detection and analysis of mutations is a very important biomarker in tumor diagnosis and prognostic prediction. Currently available prognostic indexes for ENKTCL, e.g., IPI, PINK, and PINK E, do not incorporate molecular mutations as one of the prognostic markers. Recently, three molecular subtypes, namely, MB, TSIM, and HEA subtypes are described to account for different genomic alterations profile and different EBV pattern in ENKTCL based on genomic and transcriptomic data [54]. MB subtypes are enriched for MYC-associated aberrations, mainly as MGA (a tumor suppressor) mutations and loss of heterozygosity (LOH) at the BRDT locus. TSIM subtypes are characterized by aberrations in tumor suppressors (TP53 mutation and del6q21) and immune modulators (Jak-STAT mutation/amplification and amp9p24.1/PD-L1/2 locus). Lastly, the HEA subtype is enriched for T cell gene expression and mutations in epigenetic regulators (EP300, HDAC1, and ARID1A) [54]. Interestingly, the MB subtype was associated with much poorer prognosis than the TSIM and HEA subtypes with 3-year survival rates as 38.5%, 79.1%, and 91.7%, respectively [54]. Since MB subtypes presented with MYC-associated aberrations, homoharringtonine (HHT), which is a protein translation inhibitor that exhibits an antileukemia effect through regulating MYC transcriptional expression [55], was shown to exhibit an inhibitory effect on both the MGA-knockdown cell model and the xenograft zebrafish model [54]. With the advancement of sequencing technology, featured by longer read length and faster processing time, a plethora of different molecular mutations and thus different molecular subtypes will be illustrated for prognostication and eventually therapeutic targets for ENKTCL.

From a technical point of view, when the biopsy tissue is too necrotic, particularly in ENKTCL, and NGS testing of the biopsy specimen is unfeasible, cell-free circulating tumor DNA (ctDNA) could possibly be an important complementary source for NGS testing in patients with NKTCL. CtDNA results from tumor cell apoptosis and necrosis with a characteristic peak at 166 bp [56]. Li et al. successfully used ctDNA to identify mutations verified in NKTCL tumors and achieved satisfactory sensitivity (72.4%) [57]. Furthermore, additional somatic mutations can be detected in ctDNA due to tumor spatial heterogeneity. Because of its ease of repeatability and simplicity, ctDNA is likely to become a convenient tool for NKTCL diagnosis and assessment in the clinical and research setting in the future. However, attention must be paid to several important aspects in handling ctDNA. For instance, single centrifugation alone cannot effectively remove all the nucleated cells in plasma, and this can potentially lead to the contamination of ctDNA. Chiu et al. reported that double centrifugation (1600× *g* for 10 min, then 16,000× *g* for 10 min) is the best and most cost-effective method to confidently obtain cell-free plasma [58]. Another method is centrifugation followed by filtration, but this method is more expensive [58]. Plasma should be stored at −20 °C or −80 °C if ctDNA is not extracted immediately. Chan et al. also demonstrated that ctDNA will become more fragmented if the plasma undergoes freezing-thawing more than twice [59]. The choice of anticoagulant used in blood collection is also an essential element. Beutler et al. demonstrated that heparin could inhibit PCR, which makes it an unfavorable choice for samples that are to undergo NGS [60]. Lam et al. showed that contaminated DNA content was lower in plasma samples collected in ethylenediaminetetraacetic acid (EDTA) tubes than in heparin or citrate tubes, likely because EDTA causes less leucocyte necrosis or apoptosis when samples are stored for 24 h. Thus, EDTA is suggested to be a better anticoagulant for blood samples that may be processed for plasma DNA analysis up to 6 h after venesection [61].

## 7. Molecular Pathogenesis of ENKTCL and Its Implication in Personalized Treatment of ENKTCL

In the past, ENKTCL was treated using conventional chemotherapy and radiotherapy. There are more treatment choices for ENKTCL nowadays with the increased understanding of the pathogenesis of the disease, and novel agents are now available targeting different molecular pathways discovered in ENKTCL. Such advances are important since conventional chemotherapy may not achieve long-term remission in advanced-stage disease.

### 7.1. Brentuximab Vedotin (Anti-CD30 Antibody-Drug Conjugate)

CD30 (TNFRSF8) is a glycoprotein of the tumor necrosis factor receptor (TNFR) expressed on activated B, T, and NK cells. CD30 can activate the NF-κB signaling pathway and the mitogen-activated protein kinases (MAPKs) to modulate cell growth, proliferation, and apoptosis.

Between 31.2% and 47.3% of patients with ENKTCL are positive for CD30 [62], but the relationship between CD30 expression and the prognosis or clinical characteristics of ENKTCL remains unclear. The expression of CD30 was shown to be significantly associated with longer OS in a study of 72 patients with NKTCL who were treated with non-anthracycline-based chemotherapy [63]. Another study suggested that CD30 was an independent prognostic factor for OS and PFS [64].

A multicenter, phase II study of brentuximab vedotin was conducted in 33 patients with relapsed/refractory CD30-positive non-Hodgkin lymphoma, seven of whom had ENKTCL. Brentuximab vedotin was administered once every 3 weeks. The overall response rate for patients with ENKTC was 29%, with one patient obtaining complete remission and another achieving partial remission; the response lasted for more than one year in both patients [65].

Further studies are needed for the assessment of the efficacy of brentuximab vedotin, particularly in combination with chemotherapy, in the treatment of NK/T-cell lymphoma.

### 7.2. Immune Checkpoint Inhibitors (PD1 and PDL1 Inhibitors)

PD-L1 expression is higher in patients with EBV-positive compared with EBV-negative non-Hodgkin lymphoma [66], and the expression of PD-L1 in NKTCL ranges from 39% to 100% [67,68,69,70,71].

Programmed cell death protein-1 (PD-1)/PD-L1 pathways act as a “brake” to control and maintain immune tolerance within the tumor microenvironment by decreasing T cell activation, proliferation, cytokine secretion, and survival [72]. An association and interactions between EBV and the PD-1/PD-L1 pathway has been noted in different malignancies [73,74,75]. Moreover, EBV-driven LMP1 upregulated PD-L1 expression by activating the MAPK/NF-κB pathway [76]. There was overexpression of PD-L1 in both serum and tumor specimens from patients with ENKTCL, and this carried adverse prognostic impacts [69,76].

Studies using PD-1/PD-L1 inhibitors have shown promising results. Kwong et al. showed that PD-1 blockade with pembrolizumab was useful for the treatment of relapsed or refractory ENKTCL. Seven patients with relapsed or refractory ENKTCL (1 with stage I and 6 with stage IV disease) who had previously received L-asparaginase-based regimens were treated with pembrolizumab. The pembrolizumab dose was 2 mg/kg every 3 weeks in all but one patient, who received pembrolizumab every 2 weeks. The ORR was 100%; five patients (71.4%) achieved CR, and two even achieved molecular remission. The five patients who achieved CR were still in remission after a median follow-up of 6 months [77].

Li et al. also reported the efficacy of pembrolizumab in a series of seven patients with refractory/relapsed NKTCL [78]. Pembrolizumab was administered at a dose of 100 mg every 3 weeks for a median of four cycles (range 2–18). The ORR was 57.1%, with two patients obtaining CR (28.6%) and two achieving a partial response (28.6%). The response duration was 4.1 months, PFS 4.8 months, and OS 5.0 months. The combination of immune checkpoint inhibitors with brentuximab vedotin could also potentially increase antitumor activity [79].

It is clinically important to identify biomarkers that identify patients with a higher chance of response to immune checkpoint inhibitors. PD-L1 structural rearrangement (PD-1MUT) level is a potential biomarker for clinicians to choose anti-PD-1 therapy for patients with NKTCL [80]. Clinicians may consider selective use of immune checkpoint inhibitors based on PD-1MUT level so as to minimize potential side effects.

### 7.3. Daratumumab (CD38 Monoclonal Antibody)

Daratumumab, a CD38 monoclonal antibody, was approved as therapy for patients newly diagnosed or relapsed with multiple myeloma [81,82,83,84]. CD38 may also be a novel target for the treatment of patients with NKTCL since CD38 expression was found in 95% of tumor samples in a study of 94 patients with NKTCL. Clinical data suggests that CD38 may also be a novel prognostic biomarker in patients with NKTCL [85].

Daratumumab was used as monotherapy in a phase 2 study in 32 patients with ENKTCL. Daratumumab was given in 28-day cycles at a dosage of 16 mg/kg intravenously once weekly for cycles one and two, every other week for cycles three through six, and every 4 weeks thereafter until progression or intolerable toxicity. The results showed an ORR of 25.0%, although CR was not achieved in the study. The median OS and PFS were 141 days and 53 days, respectively, with a median follow-up of 10.2 months [86]. Daratumumab monotherapy may not be adequate to treat patients with aggressive features as it has modest activity as a single agent against NKTCL. Combination with other chemotherapy will be the future direction of clinical trials.

### 7.4. CC Chemokine Receptor 4 (CCR4)

Chemokines can promote progression, metastasis, and neovascularization of EBV-related hematologic malignancies [87,88]. Levels of chemokine (CC motif) ligand (CCL) 17 and CCL22 were significantly elevated in the sera of patients with ENKTCL. CCR4 is the receptor for CCL17 and CCL22 and was detected in the cell lines and tumor tissues of patients with ENKTCL [89].

Mogamulizumab is a humanized monoclonal antibody shown to be effective in killing CCR4-positive cells through the antibody-dependent cellular cytotoxicity, and to inhibit the growth of EBV-positive NK-cell lymphomas in murine models [88]. This suggests that blocking the interaction of CCR4 with its ligands CCL17 and CCL22 may have potential as a therapeutic option for ENKTCL.

### 7.5. Signal Transduction Pathway Targets

#### 7.5.1. Platelet-Derived Growth Factor (PDGF) and PDGFR Antagonists

The platelet-derived growth factor receptor α (PDGFRα) is a receptor tyrosine kinase that mediates the interaction between external cell signals and the intracellular PI3K-AKT and JAK-STAT3 pathway proteins [90]. Abnormal expression of PDGFR and aberrations in the PDGF pathway are common in the oncogenesis of different types of tumors [91,92,93].

High PDGFRα expression is a marker of a poor prognosis (lower OS and PFS) in patients with ENKTCL [94], and PDGFR is correlated with reduced PFS [15]. PDGFR antagonists have been shown to induce G1 cell cycle arrest and inhibit cell proliferation in ENKTCL cell lines in vitro [15,95]. The results could indicate a potential role for small molecular tyrosine kinase inhibitors targeting the PDGF pathway in ENKTCL.

#### 7.5.2. Janus Kinase-Signal Transducer and Activator of Transcription (JAK-STAT) and JAK-STAT Inhibitors (WP1066, CP-690550, AG490, Tofacitinib, and Chidamide)

Both in vitro studies and gene expression profiling data revealed recurrent mutations in Janus kinase (JAK)-signal transducer and activator of transcription (STAT) pathway genes in ENKTL [21,96], leading to aberrant pro-proliferative signaling. It was found that 75.0% of ENKTCL biopsy specimens showed expression of pSTAT3, and pSTAT3 expression was correlated with Ki67 overexpression. The selective inhibitor, WP1066, induced apoptosis of ENKTCL cells via STAT3 suppression [96]. A functional *JAK3* mutation associated with JAK-STAT activation was present in 23 of 65 (35.4%) patients with ENKTCL. Interestingly, JAK3 was shown to mediate a functional switch of EZH2 from histone methyltransferase to a transcriptional coactivator in ENKTCL and upregulates a set of genes involved in DNA replication, cell cycle regulation, biosynthesis, cell stemness, and invasiveness. [97]

The use of pan-JAK inhibitor CP-690550 and JAK2 inhibitor AG490 resulted in dose-dependent reduction of pSTAT5 and pSTAT3 together with increased apoptosis in ENKTCL cell lines [98,99]. Moreover, the JAK1/3 inhibitor, tofacitinib, induces G1/S arrest and inhibits cell growth in STAT3-mutant ENKTCL cells [100].

An ongoing phase 2 clinical trial (NCT03598959) is investigating the efficacy and safety of a combination of tofacitinib and chidamide in patients with relapsed or refractory ENKTCL.

#### 7.5.3. NF-κB Signaling and Proteosome Inhibitors

One of the hallmarks of ENKTCL is the persistent activation of the NF-κB signaling pathway. It is thought that activation of NF-κB signaling may account for the transformation of EBV-infected cells into ENKTCL cells [101,102].

Proteasome inhibitors have latency-reversing properties that can reactivate EBV into the lytic cycle [103]. They will then help the immune system or anti-EBV drugs to kill the EBV-infected tumor cells (i.e., the “shock and kill theory”) [104].

Bortezomib is a first-generation proteasome inhibitor. The combination of bortezomib and GIFOX (gemcitabine, ifosfamide, oxaliplatin) chemotherapy resulted in an ORR of 43% and a median PFS of 4.3 months in seven patients with newly diagnosed ENKTCL, four of whom had advanced-stage disease [105].

Through activation of NF-κB pathway, LMP-1 was shown to upregulate CD25 expression in ENKTCL, which would then induce resistance to asparaginase in ENKTCL cell lines [101]. Thus, the combination of proteasome inhibitors and asparaginase-based chemotherapy may further improve the treatment responses of patients with relapsed/refractory ENKTCL.

### 7.6. Histone Acetylation and Histone Deacetylase Inhibitor (Chidamide)

Histone deacetylase (HDAC) inhibitors can induce different cytotoxic effects in cancer cells by histone acetylation of candidate tumor suppressors to initiate gene transcription [106]. Chidamide is a novel oral agent belonging to the benzamide class and is a subtype-selective inhibitor of HDAC 1, 2, 3, and 10. It also interferes with PI3K/Akt/mTOR and MAPK signaling and inhibits cell proliferation [107].

In a prospective phase 2 trial, chidamide monotherapy was studied in 15 patients with relapsed/refractory ENKTCL. Four of the 12 evaluable patients (33%) obtained CR with a median follow-up of 3.7 months; all CR patients remained disease-free at 6.9 months [107].

### 7.7. Chimeric Antigen Receptor T-Cell (CART) Therapy

Chimeric antigen receptor T-cell (CART) therapy is a new form of immunotherapy and has shown success in the treatment of relapses or refractory acute lymphocytic leukemia, diffuse large B-cell lymphoma, and multiple myeloma [108,109,110,111,112,113,114,115]. There are currently limited data on the use of CART therapy for ENKTCL, but the results of a phase 1 clinical trial of CD30 CART therapy for patients with advanced CD30-expressing lymphomas are pending (NCT03049449). It may be a promising treatment option.

### 7.8. Apoptotic Pathways and Other Tumor Suppressor Genes

Deregulation of apoptotic pathways is another mechanism by which ENTKL arises and progresses. Overexpression of surviving and mutations of TP53 conferred a survival advantage on the ENKTL cells [116]. Mutated TP53 was detected in 40% of cases in one study in China [117]. Mutated TP53 correlates with advanced stage presentation and poor prognosis in ENKTCL [118].

In addition, deletion at chromosome region 6q21–23, the most common cytogenetic aberrations have been implicated in the loss of several tumor suppressor genes including PRDM1, FOXO3, HACE1, and PTPPK [116]

At the time of writing this manuscript, no target therapies acting against the aforementioned pathways and genes have been developed in the treatment of ENKTCL.

A summary of novel agents in development or being investigated for ENKTCL is shown in Table 2 [15,65,77,86,88,95,100,105,107].

## 8. Conclusions

ENKTCL is an aggressive malignancy with unique epidemiological, histological, molecular, and clinical characteristics. The use of high-throughput sequencing has allowed us to gain more insight into the molecular mechanisms of ENKTCL, which largely involves chromosome deletion and aberrations in the JAK/STAT and PD-1/PD-L1 pathways, as well as mutations in tumor suppressor genes. This information has provided new perspectives on immunotherapy and targeted therapy within the spectrum of ENKTCL treatment. These molecular findings could potentially revolutionize the traditional chemoradiotherapy approach, which is known to be associated with significant toxicity. The use of immunotherapy in combination with other mechanism-based targeted therapies is a promising strategy to treat ENKTCL.

## Figures and Tables

**Table 1:** Summary of mutated genes from five NGS studies in patients with ENKTCL [19,20,21,27,28]. Only mutated genes that were identified in ≥2 patient samples/cell lines in the reference studies are listed in the table. Tiers of gene mutations are classified according to guideline from Association of Molecular Pathology, American Society of Clinical Oncology, and College of American Pathologists [53]. Only *DDX3X* and *TP53* mutations have been shown to have prognostic impact.

Mechanism of Lymphomagenesis	Gene	Tier ^#^	Mutations	Reference
Cell adhension	*CHST10*	III	missense	[19]
	*SDK2*	III	missense	[21]
	*PCDH15*	III	missense	[19]
	*FLRT3*	III	missense	[19]
Microtubule-related	*DNAH11*	III	missense	[19]
	*MAST4*	III	missense	[20]
	*DCLK1*	III	missense, nonsense	[21]
	*DNAH14*	III	frameshift insertion, missense	[21]
Tyrosine kinase	*MAP2K1*	II	missense	[19]
	*STAT3*	I	missense	[21,28]
	*Jak3*	I	missense	[28]
	*STAT5B*	I	missense	[28]
	*ROS1*	II	missense, nonsense	[20,21]
	*FGFR2*	II	missense	[21]
	*JAK1*	I	missense	[21]
	*KRAS*	I	missense	[21]
	*PTK2B*	II	missense	[21]
	*TYK2*	III	missense, frameshift insertion	[21]
	*MAP3K5*	III	missense	[19]
	*MAP2K1*	II	missense	[19]
G protein-coupled receptor	*SMO*	II	missense	[20]
	*TSHR*	III	missense	[21]
Regulator of signaling pathway	*RGS7*	III	missense	[19]
	*AKAP6*	III	missense	[21]
	*AMER1*	II	missense	[20]
	*RASGRP2*	III	missense	[20]
	*LRRK2*	III	missense	[20]
	*PLCG1*	III	missense, frameshift deletion	[21]
	*USP34*	III	missense	[21]
	*LRP1B*	III	missense	[19]
	*DGKD*	III	missense	[19]
	*RGS7*	III	missense	[19]
	*TIAM1*	III	missense	[19]
	*SHC4*	III	missense	[19]
	*PRKD1*	III	missense	[19]
Tumor suppressor gene	*Tp53*	I	missense, nonsense	[19,20,21,28]
	*APC*	II	missense	[21]
	*LRP1B*	III	missense	[19]
	*BAP1*	II	missense	[21]
	*NEK4*	III	missense	[21]
	*ZFHX3*	III	missense	[19]
Chromatin modifying/remodelling gene	*EP300*	II	missense	[19]
	*BCOR*	I	missense, nonsense, frameshift deletion	[20,21,28]
	*MLL2*	I	missense, nonsense	[21,27]
	*BAZ1A*	III	missense	[20]
	*MLL3*	II	missense	[20]
	*TET2*	I	nonsense, missense	[20]
	*ARID1A*	II	missense, frameshift insertion	[21]
	*BAZ2B*	III	missense	[19]
Calcium ion binding	*FAT4*	III	missense	[19]
	*FAT3*	III	missense	[19]
Collagen binding and myosin binding	*USH2A*	III	nonsense, missense	[21]
DNA-binding transcription factor	*BCL11A*	III	missense	[21]
	*MGA*	II	missense	[18,19,20]
	*TBP*	III	frameshift deletion	[20]
	*PRDM16*	III	missense	[21]
	*ZFP28*	III	missense	[19]
	*ZNF626*	III	missense	[19]
Maintenance of structural integrity of mitotic centromeres	*FRY*	III	missense	[21]
Extracellular matrix-related	*MXRA5*	III	missense, nonsense	[21]
	*ADAMTS20*	III	missense	[20]
	*LAMA5*	III	missense	[20]
	*LAMA3*	III	missense	[21]
	*SSC5D*	III	missense	[21]
	*IMPG2*	III	missense	[19]
	*RELN*	III	missense	[19]
	*COL4A1*	III	missense	[19]
	*LAMA1*	III	missense	[19]
DNA repair	*BRCA2*	II	missense	[21]
	*SETD2*	II	missense	[20]
	*MCMDC2*	III	missense	[21]
RNA helicase	*DDX3X*	I	missense	[19,28]
Cytoskeleton struture-related	*MSN*	III	missense	[28]
	*EPPK1*	III	missense, frameshift deletion	[20]
	*DST*	III	missense	[21]
	*PPP1R9A*	III	missense	[19]
	*PLS3*	III	missense	[19]
Cytoplasmic trafficking	*ANKRD50*	III	missense	[21]
	*EXOC4*	III	missense	[21]
Apoptosis	*CASP8*	II	missense, nonsense	[21]
Growth factor -related	*FGF10*	III	missense	[21]
Tyrosine kinase adaptor	*GAB4*	III	missense	[21]
Interleukin receptor	*IL6R*	III	missense	[21]
	*IL23R*	III	missense	[19]
Regulation of cell polarity	*MPP7*	III	missense	[21]
Cell cycle control	*SDE2*	III	missense	[19]
Miscellaneous	*FSIP2*	III	missense	[21]
	*FUT4*	III	missense	[20]
	*HLA-A*	III	nonsense	[20]
	*LRP2*	III	missense	[20]
	*MALAT1*	III	missense	[20]
	*C17orf104*	III	missense	[21]
	*CHMP2B*	III	missense	[21]
	*EPRS*	III	missense	[21]
	*IARS2*	III	missense	[21]
	*MPDZ*	III	missense	[21]
	*MUC5B*	III	missense	[21]
	*NCKAP5*	III	missense	[21]
	PDE4DIP	III	missense, frameshift deletion	[21]
	*PRKACA*	III	missense	[21]
	*PRPF4B*	III	missense	[21]
	*STOX2*	III	missense	[21]
	*STXBP5*	III	missense	[21]
	*WDR17*	III	missense	[21]
	*TTN*	III	missense	[19]
	*CHPF2*	III	missense	[19]
	*MYH3*	III	missense	[19]
	*PCLO*	III	missense	[19]
	*ATP10B*	III	missense	[19]
	*KBTBD4*	III	missense	[19]
	*MGAM*	III	missense	[19]
	*MAGEB6*	III	missense	[19]
	*ATRNL1*	III	missense	[19]
	*HYDIN*	III	missense	[19]
	*NLGN4X*	III	missense	[19]
	*CPS1*	III	missense	[19]
	*CSMD2*	III	missense	[19]
	*TCN1*	III	missense	[19]

#Tier I represents variants of strong clinical significance. Tier II represents variants of potential clinical significance. Tier III represent variants of unknown clinical significance.

**Table 2:** Novel agents and their targets in the treatment of ENKTCL.

Drug	Novel Target	Study Population	Outcome	Reference
Brentuximab	CD30	Multicenter phase 2 study in relapsed/refractory patients	ORR 29% CR 14% PR 14%	[65]
Daratumumab	CD38	Multicenter phase 2 study in relapsed/refractory patients	ORR 25%, median response duration 55 days	[86]
Pembrolizumab	PD1, PDL-1 inhibitor	Relapsed/refractory patients who failed L-asparaginase based regimen (1 with stage 1, 6 with stage IV)	ORR 100%, CR 71.4%	[77]
PDGFR antagonist	Inhibit proliferation of ENKTCL cell lines in vitro	Preclinical		[15,95]
Mogamulizumab	Monoclonal antibody of CCR4	Preclinical		[88]
Tofacitinib	JAK1/3 inhibitor	Relapsed/refractory patients	Data not yet published	[100]
Bortezomib	NF-κB signaling pathway “Shock and kill theory” in EBV infected cell lines	Bortezomib + GIFOX in newly diagnosed ENKTCL	ORR 43% and median PFS of 4.3 months	[105]
Chidamide	Selective inhibitor of HDAC 1, 2, 3 and 10	Phase 2 trial in relapsed/ refractory ENKTCL	33% CR with median follow-up of 3.7 months	[107]

CCR4, CC chemokine receptor 4; CR, complete remission; EBV, Epstein–Barr virus; ENKTCL extranodal natural killer T-cell lymphoma; GIFOX, gemcitabine, ifosfamide, oxaliplatin; HDAC, histone deacetylase; JAK, Janus kinase; NF-κB, nuclear factor κB; ORR, overall response rate; PD-1, programmed cell death protein-1; PD-L1; programmed cell death ligand-1; PFS, progression-free survival; PR, partial remission.

## Data Availability

Not applicable.
